# Multidrug-resistant organisms in lung transplant: a narrative review

**DOI:** 10.1097/MOT.0000000000001066

**Published:** 2023-03-28

**Authors:** Sabrina Congedi, Paolo Navalesi, Annalisa Boscolo

**Affiliations:** a Department of Medicine (DIMED), University of Padua; b Institute of Anaesthesia and Intensive Care, Padua University Hospital, Padua, Italy

**Keywords:** bacteria, lung transplantation, multidrug or multidrug resistant

## Abstract

The purpose of this narrative review is presenting the current knowledge of multidrug-resistant (MDR) pathogens in lung transplant recipients, considering both Gram-positive and Gram-negative bacteria.

**Recent findings:**

Overall prevalence of Gram-negative pathogens has increased remarkably (4.33/1000 recipient-days) in solid organ transplant recipients, while the prevalence of Gram-positive bacteria seems to be decreasing (0.20 cases/100 transplant-years). In lung transplant, the prevalence of postoperative infections due to MDR-GN bacteria has been assessed between 31 and 57%, and the incidence of carbapenem-resistant Enterobacterales is between 0.4 and 20%, with a related mortality up to 70%. MDR *Pseudomonas aeruginosa* is common in lung transplant recipients with cystic fibrosis and may contribute to bronchiolitis obliterans syndrome. The prevalence of MDR Gram-positive bacteria is around 30% (predominantly Methicillin-resistant *Staphylococcus aureus* and Coagulase-negative staphylococcus).

**Summary:**

Survival after lung transplant, although lower than in other SOT, is increasing and currently at 60% at 5 years. This review highlights the potential clinical and social burden of postoperative infections in lung transplant recipients, and confirmed that a PI due to MDR bacteria negatively affects survival. A prompt diagnosis, prevention and management of these MDR pathogens should remain the cornerstone for higher goals of care.

## INTRODUCTION

One of the main challenges for modern medicine is preventing, containing and treating the outbreak of the emerging pathogens with antibiotic resistances [1]. Actually, the prevalence of antibiotic resistance strains of bacteria is increasing worldwide, representing a major challenge especially for higher-risk subgroups of patients, such as solid organ transplant (SOT) recipients [2]. SOT recipients represent a patient category prone to develop infections, especially caused by multidrug-resistant (MDR) pathogens [1,2]. The risk of significant posttransplant infections is considerable and probably due to high-dose immunosuppressants, broad-spectrum antibiotics, precolonizations, difficult or prolonged weaning, need of tracheostomy and prolonged hospitalization [3^▪^,^4^]. Moreover, in the last decade, several articles confirmed that posttransplant infections, occurring after SOT, are related to worse outcomes, even in lung transplant recipients [5–7]. Posttransplant infections seem to be an independent risk factor of mortality and re-transplantation in the SOT population, with a prevalence of 42% [8^▪▪^]. Noteworthy lung transplant recipients experienced posttransplant infections more frequently than other SOT patients, with an incidence of MDR bacteria ranging between 31 and 57% [9]. Moreover, recent data showed that lung transplant recipients with perioperative MDR bacterial infection recorded higher in-hospital mortality, estimated to be up to six times greater than in non-MDR lung transplant controls [10^▪^]. In fact, recipients undergoing lung transplant, for indications other than cystic fibrosis, have been shown to develop frequently perioperative pneumonia (35%), and often caused by MDR bacteria [8^▪▪^,11^▪^,12^▪^]. However, the prevalence seems different between Gram-positive and Gram-negative bacteria [13]. Bartoletti *et al.*[13] have investigated the epidemiological trend of bacteria in SOT in the last decade and showed as the prevalence of Gram-negative pathogens has increased remarkably (4.33 per 1000 recipient-days), while the prevalence of Gram-positive bacteria has not (0.20 cases per 100 transplant-years). Noteworthy, the research ‘gram negative bacteria’ and ‘LT’, picking up ‘AND’ as Boolean operator, on PubMed Network (https://pubmed.ncbi.nlm.nih.gov), produced more than 600 results from 1992 to 2022, and half of these articles have been published in the last 6 years. Assuming an evident need of updating the body of knowledge of the infectious complications, the current review emphasizes the major data available in literature regarding MDR-related infections in lung transplant recipients, considering both Gram-positive and Gram-negative antibiotic-resistant pathogens. 

**Box 1:**
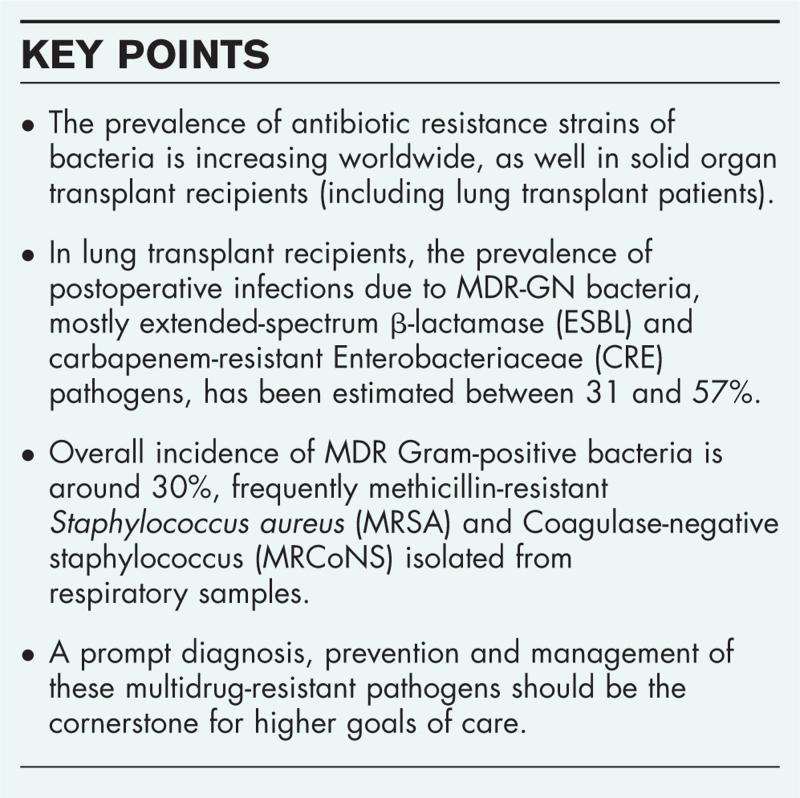
no caption available

## MATERIALS AND METHODS

This narrative review discusses the incidence of bacteria with antibiotic resistance in lung transplant recipients. Scientific articles published, in the last 15 years, on MEDLINE, EMBASE and the Cochrane Library, were considered. The terms ‘MDRO’, ‘Multidrug Resistant Organisms’, ‘bacteria’, ‘pathogens’, ‘lung transplant’, ‘gram-positive’, ‘gram negative’, ‘methicillin-resistant S. aureus’, ‘MRSA’, ‘Coagulase-negative staphylococcus’, ‘CoNS’, ‘ampicillin-resistant Enterococcus species’, ‘extended-spectrum β-lactamase’, ‘ESBL’, ‘carbapenem-resistant Enterobacteriaceae’, ‘CRE’, ‘Carbapenem-resistant gram negative bacteria’, ‘extensively drug resistant’, ‘XDR’, ‘pan-drug resistant’, ‘PDR’ were used for the research; ‘AND’ was used as a Boolean operator. Moreover, in order to identify relevant studies, the cited references of the selected articles were reviewed. The authors’ personal collections of literature were also browsed. Articles, which emerged from the mentioned research, were selected for consideration in this manuscript according to their relevance for the topic, as judged by the authors (AB, SC, PN).

In accordance with the literature, Gram-negative bacteria were defined as extended-spectrum β-lactamase (ESBL) pathogens when able to hydrolyze extended spectrum cephalosporins; carbapenem-resistant Enterobacteriaceae (CRE) when having phenotyping tests for carbapenemase production; MDR pathogens when resistant to at least one agent in at least three antimicrobial classes [11^▪^,^14^^▪▪^,^15^–^17^]. Likewise, a Gram-positive antibiotic-resistant bacteria was defined as methicillin-resistant *Staphylococcus aureus* (MRSA), Coagulase-negative staphylococcus (CoNS), ampicillin-resistant Enterococcus species. We also investigated data available in literature regarding extensively drug resistant (XDR) and pan-drug resistant (PDR) pathogens. XDR was defined as susceptibility of a bacteria to only one or two categories and XDR as a pathogen with no-susceptibility to all agents in all antimicrobial categories [11^▪^].

## MULTI-DRUG RESISTANT GRAM-NEGATIVE BACTERIA

### Extended-spectrum β-lactamase gram-negative bacteria

ESBL Gram-negative bacteria phenotype is characterized by resistance to penicillins and cephalosporins and susceptibility to carbapenems and their prevalence is increasing worldwide [2]. The ESBL are exogenous genes located in mobile genetic elements called plasmids that can be acquired by Enterobacteriaceae (such as *Escherichia coli* and *Klebsiella pneumoniae*), *Pseudomonas aeruginosa* and Acinetobacter spp., while Enterobacter spp., *Citrobacter freundii* and *Morganella morganii* usually express inducible chromosomal beta-lactamases (ampC) [18–20]. In SOT patients, bacterial infections caused by ESBL-producing Enterobacteriaceae ranges between 2 up to 10% and mostly occurred in the early posttransplant period [9,21–23]. Unfortunately, data are still conflicting in the lung transplant population. Recently, Boscolo *et al.*[10^▪^], investigating potential risk factors of perioperative MDR and/or ESBL-GN isolations, reported an ESBL-GN bacteria prevalence around 5% (nine out to 153 lung transplant recipients), predominantly from respiratory samples, and a similar survival rate between recipients affected by ESBL and MDR-GN pathogens.

Oriol *et al.*[24] investigated the cause of bloodstream infections among SOT, also including several lung transplant recipients, and reported an increased rate of MDR-GN, mostly ESBL-producing strains, during the 10-year of the observation (up to 34% of cases), and principally due to *K. pneumoniae*. Noteworthy, mortality associated with infection owing to ESBL-producing strains ranged between 8 and 26% [22,23]. With regards to bowel colonization by ESBL or ampC-producing Enterobacteriaceae, it represents one of the most important risk factors of perioperative and postoperative infection in SOT, and even in lung transplant recipients [25]. In fact, colonization seems proportional to bloodstream bacteria dissemination [25]. Finally, Penã *et al.* listed specific clinical characteristics, frequently occurring in lung transplant population, as potential risk factors for ESBL colonization: SOT, ICU admission and clinical severity, advanced age, central catheters or other intravascular devices, need of mechanical ventilation, renal replacement therapy, parenteral nutrition, urinary catheterization and previous antibiotic treatment [26,27].

### Carbapenem-resistant gram-negative bacteria

Gram-negative bacteria represent a major concern due to the alarming spread of carbapenem resistance worldwide [28], because carbapenem-resistant Gram-negative bacteria (CR-GNs) are difficult to treat and are often correlated to significant morbidity and mortality, particularly among SOT recipients [29,30]. After the meeting of the European Antimicrobial Resistance Surveillance System in 2010 and the workshop at the Dutch National Institute for Public Health and the Environment in 2013, the so-called ‘EuSCAPE project’ was developed with the aim to collect comprehensive data about CR-GN epidemiology, and the diffusion of this bacterial strain, predominantly Enterobacterales (CRE) in Europe [30]. The survey suggested a continuous and growing CR-GNs spreading in European hospitals and confirmed a ‘shift’ from a ‘single hospital outbreak’ to a predominance of ‘regional and inter-regional spread’ [30].

This worrisome trend reflects the rapid expansion of CRE-GNs in SOT patients. In the last Italian nationwide survey, enrolling 887 SOT recipients, the incidence of CRE-GN bacteria was 0.63 per 1000 recipient-days and carbapenem resistance was particularly frequent among *Klebsiella* spp. isolates (49.1%) [31]. In lung transplants, the prevalence has been assessed between 0.4 up to 20% [9]. CRE infection commonly occurs in SOT in the initial posttransplant period (on average 11–36 days) [31], but data regarding exclusively lung transplant recipients are not available. Infections due to CRE are usually bloodstream infections (BSI), including catheter-related BSI, pneumonia, urinary tract infection (UTI) and intra-abdominal infections [9]. The CRE-associated mortality rate in SOT is consistent (up to 70%), as confirmed by The American Society of Transplantation Infectious Diseases Community [15,29,31,32]. Likewise, Yanik Yalçin *et al.*[33] confirmed that the above-mentioned mortality rate was even higher in lung transplant recipients with CRE infections. With regards to MDR *P. aeruginosa*, a pretransplant colonization of the respiratory tract is especially common in lung transplant recipients with cystic fibrosis (CF), with a prevalence of 50% that may increase up to 75% after lung transplant [34]. On the contrary, *P. aeruginosa* is the leading cause of hospital-acquired pneumonia (HAP) occurring after lung transplant (up to 25% of cases) [35]. Finally, several items, such as prolonged hospital stay, lung graft and previous hospitalization have been identified as important risk factors for CRE colonization and/or infection in SOT and similarly in lung transplants [10^▪^,^31^].

### XDR and PDR bacteria

MDR GN bacteria isolation after transplantation is increasing, and no extended data are available regarding infections by XDR and PDR pathogens in lung transplant recipients. Although this, some findings could be extrapolated from previous retrospective studies and one case report [33,36–38]. Yanik Yalçin *et al.* [33] enrolling 164 SOT recipients from 11 Turkish hospitals, described 171 episodes of XDR GN bacteraemia, 63.7% of whom within the first year after surgery. The most common XDR pathogen, identified in one-third of the lung transplant cohort of patients, was *K. pneumoniae* with a rate of 7-day mortality around 36% [33]. According to these findings, 63.7% of XDR GN bacteria occurred in the first year after transplantation and the highest rate of early-onset bacteraemia was in lung transplant patients [33]. PDR and XDR bacteria in the SOT population are mostly gram-negative pathogens and often occur in lung transplant due to cystic fibrosis [36,38,39]. This inference was confirmed by Winstead *et al.*[38], studying 44 patients, requiring lung transplant due to CF, and chronically infected by PDR *P. aeruginosa*. Indeed, lung transplant recipients with CF are probably more susceptible to develop XDR or PDR colonizations and/or infections due to prolonged hospitalizations, recurrent antimicrobial therapies, exposure to multiple invasive procedures or to indwelling devices. According to the 2017 International Society for Heart and Lung Transplantation (ISHLT) Thoracic Transplant Registry Report, patients with CF comprised 23% of 36.046 bilateral lung transplant worldwide [40]. Finally, several authors, investigating the survival rate in CF patients with higher resistance bacterial infections, found no differences in the overall 90-day and 1-year survival rate between CF recipients with high-resistance bacterial infection and those without [36,39].

## MULTIDRUG-RESISTANT GRAM-POSITIVE BACTERIA

Bartoletti *et al.*[13] showed that the prevalence of Gram-positive bacteria in SOT recipients is in decline (0.20 cases per 100 transplant-years). However, the overall incidence of Gram-positive bacterial infections continued to be higher in lung transplant recipients than in other SOT patients [9]. Tebano *et al.*[4] retrospectively analysed a cohort of 176 lung transplant patients, collecting respiratory samples, and reported an overall incidence of MDR GP bacteria of 32.7%. MRSA and MRCoNS pathogens have been more frequently identified (in 19.6 and 11.4% of cases, respectively). Although other studies reported different epidemiological data, MRSA remains the most frequent pathogen in lung transplant recipients [11^▪^,^41^]. Paglicci *et al.*[11^▪^], enrolling 96 lung transplant recipients, reported that one-third of participants developed a bacterial colonization of the lower respiratory airways within the first month, and in almost 12% of cases was owing to MDR bacteria (30% MRSA colonization). Similar epidemiological results were described by Shields *et al.* [41]. *S. aureus* colonization was described in 26% of lung transplant patients, of whom 45% were positive for MRSA [41]. In ‘The Swiss Transplant Cohort Study (STCS)’, 1527 SOT were included and, through the analysis of different sites of microbiological sampling, only 20% experienced an enterococcal event (47% infections, 52.3% colonizations). However, enterococcal colonization rates were higher in lung transplant recipients [0.33/person-year, 95% confidence interval (95% CI) 0.24 - 0.44] than in other SOT recipients and *Enterococcus faecium* was the predominant species in case of infection (38.9%) [42]. Likewise, Lian *et al.*[43], enrolling 51 lung transplant recipients, showed that both Gram-positive and Gram-negative bacteria were mostly (68.12%) isolated in the early stage of lung transplant. Enterococcus and Staphylococcus were the most prevalent bacteria revealed on bronchoalveolar lavage fluid, but no data were available on antibiotic sensibility [43].

## COLONIZATION AND INFECTIONS: THE STATE OF THE ART

Data available in literature are in agreement with the following features: Gram-negative bacteria, including MDR/XDR/PDR, are frequently isolated after lung transplant, while Gram-positive bacteria (mainly MRSA) record a lower incidence [44]; posttransplant infections represent one of the major cause of mortality after lung transplant [45]; and graft colonization and/or infection is very common during the first 30-day after lung transplant [46].

However, there is still a gap among the management and the risk of bacteria colonization in SOT donors and recipients. With regards to donor colonizations, several articles reported a relatively low risk of donor-recipient bacteria transmission (up to 2.9% of cases). Some authors demonstrated that organisms from the donor tracheal cultures were mostly different from those associated with early infections in lung transplant recipients [47]. Similar data have been reported for MDR and high-resistance bacteria, showing a not relevant risk of transmission to lung transplant recipients [48]. With regards to pretransplant recipient's colonization, it represents an important predictive factor for developing posttransplant infections, especially in recipients with CF [10^▪^,^49^,^50^]. However, the American guidelines for antimicrobial prophylaxis in surgery suggested the use of cefazolin for heart and lung transplantations, despite previous colonizations, and recommended adjusting antimicrobial therapy according to postoperative isolations and clinical data [51]. Likewise, the Spanish guidelines confirmed that, with the available data, it is not possible to issue recommendations concerning the surgical prophylaxis in patients colonized by CPE [1]. However, a worldwide survey, conducted in 180 lung transplant centres, surprisingly showed that almost all centres adjusted antibiotic therapies based on previous colonizations and in more than 70% of cases were prescribed antibiotics against GN-resistant bacteria, even without previous bronchial colonizations [51]. Boscolo *et al.*[10^▪^] may help to better clarify this issue: previous recipient-related, but not donor-related, colonization is an independent predictor of isolation of MDR and ESBL-GN bacteria in lung transplant recipients, but not necessarily related to a subsequent severe infection. So, the use of a ‘modified’ surgical prophylaxis, based on broad-spectrum antimicrobial drugs, seems conflicting and not necessarily associated with a lower risk of MDR or ESBL GN bacteria isolation after lung transplant [1,10^▪^,^51^].

Finally, the topic of the intestinal decolonization in SOT recipients, to prevent infections by high-resistance bacteria, is still under investigation. Nowadays, two observational studies showed no advantages in the use of different type of antibiotics (such as fluoroquinolones, oral gentamicin, oral polymyxin E) to reduce the incidence of infection with ESBL, ampC or carbapenamase-producing Gram-negative bacilli [52,53].

## CONCLUSION

Since the first lung transplant performed in 1963 at the University Hospital Mississippi by Dr Hardy and his team, the surgical procedure has been significantly improved, and a multidisciplinary approach has been developed to obtain a successful organ transplantation. According to the data provided by the last International Thoracic Organ Transplant Registry of the International Society for Heart and Lung Transplantation, close to 70 000 lung transplants have been realized in the last three decades [54^▪▪^]. Survival of lung transplant recipients, although lower than in other SOT, is increasing and it is currently at 60% at 5 years [55]. This narrative review highlights the potential clinical and social burden of posttransplant infections in lung transplant recipients and confirmed that a posttransplant infection, due to MDR GN bacteria, could be disastrous. In lung transplant recipients, posttransplant infections are a frequent cause of re-admission in ICU and are associated with higher both morbidity and mortality. In fact, our article is unable to account for all possible confounders occurring after lung transplant, but it clearly underlines the negative impact of MDR bacteria on graft success. Finally, a prompt diagnosis, prevention and management of these MDR pathogens should remain the cornerstone for higher goals of care.

## Acknowledgements

*Author contributions: equal.*

### Financial support and sponsorship

*None.*

### Conflicts of interest

*There are no conflicts of interest.*

## REFERENCES AND RECOMMENDED READING

Papers of particular interest, published within the annual period of review, have been highlighted as:

▪ of special interest
▪▪ of outstanding interest
